# Improvements in Family Member Quality of Life Following a Task-Shifted Psychosocial Intervention for Road Traffic Injury Survivors in Cameroon

**DOI:** 10.64898/2026.08.10.26360156

**Published:** 2026-08-12

**Authors:** Arole Darwin Touko, Fanny Nadia Dissak Delon, Gyscard Merlin Pola, Rasheedat Oke, Catherine Juillard, Alain Chichom Mefire, Sithombo Maqungo

## Abstract

**Background:**

Road traffic injuries (RTI) -related trauma is associated with heavy psychosocial burdens for family members involved as informal caregivers after the event. Little is known about the quality of life (QoL) experience of family members of RTI survivors in sub-Saharan Africa. This study investigated the QoL outcomes of family members after the administration of the Friendship Bench psychosocial intervention among RTI survivors in Cameroon.

**Methods:**

A pre-post study design was utilized in two trauma centers in Cameroon. The relatives underwent assessment both at baseline (Session 1) and follow-up sessions (Session 6) using the WHOQOL-BREF tool, without being exposed to any intervention. The domain scores were scaled from 0 to 100. Statistical analysis consisted in paired t-tests/Wilcoxon signed-rank tests along with effect sizes using Cohen’s d.

**Results:**

The study involved twenty-nine participants. Baseline assessment revealed overall poor quality of life across all domains, with scores in the poor range (Social = 33.33 [IQR: 25.00–47.92]; Environmental = 34.38 [IQR: 25.78–45.31]; Psychological = 45.83 [IQR: 34.38–50.00]; Physical = 46.43 [IQR: 39.29–56.25]). These scores had a statistically significant increases across all domains after the intervention (p < 0.001), accompanied by medium to large effect sizes (Cohen’s d:Social = 1.35; Environmental = 1.57; Psychological = 1.46; Physical = 0.73). The relationship type was found to predict social QoL (F(6,22) = 4.57, p = 0.004), with children of patients demonstrating the greatest gains.

**Conclusion:**

Family members of RTI survivors showed a significant improvement in QoL during the Friendship Bench intervention. These results highlight the importance of evaluating family member outcomes in task-shifted psychosocial programs in LMICs.

## Full Text Availability

The license terms selected by the author(s) for this preprint version do not permit archiving in PMC. The full text is available from the preprint server.

